# Advances in Using *Hansenula polymorpha* as Chassis for Recombinant Protein Production

**DOI:** 10.3389/fbioe.2019.00094

**Published:** 2019-05-01

**Authors:** João Heitor Colombelli Manfrão-Netto, Antônio Milton Vieira Gomes, Nádia Skorupa Parachin

**Affiliations:** Grupo Engenharia de Biocatalisadores, Instituto de Ciências Biológicas, Universidade de Brasília, Brasília, Brazil

**Keywords:** *Hansenula polymorpha*, recombinant protein, methylotrophic yeast, genomic editing, bioprocess

## Abstract

The methylotrophic yeast *Hansenula polymorpha*, known as a non-conventional yeast, is used for the last 30 years for the production of recombinant proteins, including enzymes, vaccines, and biopharmaceuticals. Although a large number of reviews have been published elucidating the applications of this yeast as a cell factory, the latest was released about 10 years ago. Therefore, this review aimed at summarizing available information on the use of *H. polymorpha* as a host for recombinant protein production in the last decade. Examples of chemicals and virus-like particles produced using this yeast also are discussed. Firstly, the aspects that feature this yeast as a host for recombinant protein production are highlighted including the techniques available for its genetic manipulation as well as strategies for cultivation in bioreactors. Special attention is given to the novel genomic editing tools, mainly CRISPR/Cas9 that was recently established in this yeast. Finally, recent examples of using *H. polymorpha* as an expression platform are presented and discussed. The production of human Parathyroid Hormone (PTH) and Staphylokinase (SAK) in *H. polymorpha* are described as case studies for process establishment in this yeast. Altogether, this review is a guideline for this yeast utilization as an expression platform bringing a thorough analysis of the genetic aspects and fermentation protocols used up to date, thus encouraging the production of novel biomolecules in *H. polymorpha*.

## Introduction

Over the years, the use of unicellular microorganisms as cell factories to obtain recombinant proteins became consolidated (Kim et al., 2015a). Recombinant DNA techniques allow the introduction of foreign genes in a host organism for the production of heterologous proteins biologically actives. Within this context, the choice of the host organism is crucial, since the functionality, solubility, and activity of the protein must be preserved during its synthesis (Vaquero et al., 2015). Yeasts are commonly used for the heterologous production of proteins, especially those that require post-translational modifications for proper folding since these modifications occur less frequently in prokaryotes.

**Figure 1:**
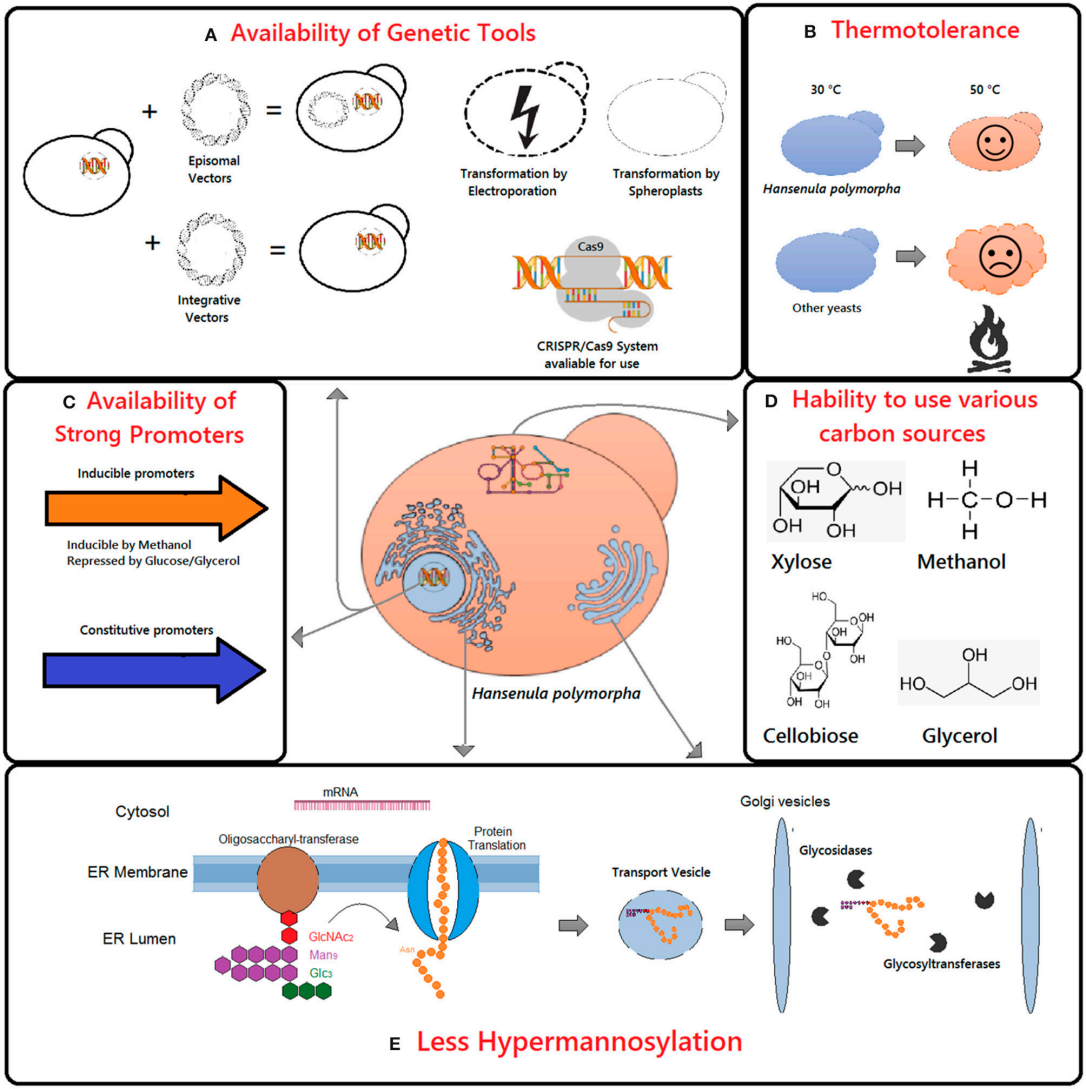
Main advantages of *Hansenula polymorpha* as chassi for recombinant protein production include the availability of genetic tools **(A,C)**, thermotolerance **(B)**, ability to use various carbon sources **(D)**, and glycosylation pattern **(E)**.

The *H. polymorpha* is commonly employed as an expression platform because of its unique characteristics (Figures 1A–E). It is thermotolerant and capable of growing at temperatures ranging from 30 to 50°C (Figure 1B). This capability is advantageous regarding mammalian protein production such as those requiring the 37°C temperature to preserve its biological activity (Van Dijk et al., 2000). Moreover, the presence of protein glycosylation pathway in *H. polymorpha* allows the production of eukaryotic recombinant proteins biologically active. Additionally, unlike other yeasts, it adds fewer sugar residues to the protein core, avoiding hyperglycosylation of recombinant proteins (Figure 1E). Finally, *H. polymorpha* is capable of using methanol as a carbon source which allowed the isolation of strong methanol inducible promoters (Figure 1C). Besides, it can utilize other carbon sources such as glycerol, glucose, xylose, and cellobiose (Ryabova et al., 2003) (Figure 1D).

Three parental strains with distinct origins of *H. polymorpha* are frequently used for recombinant protein production. The DL-1 strain (NRRL-Y-7560; ATCC26012) was isolated and characterized from soils samples (Levine and Cooney, 1973). The CBS4732 strain (CCY38-22-2; ATCC34438, NRRL-Y-5445) was isolated in irrigated soils in Pernambuco, Brazil (Morais and Maia, 1959). These two strains are mostly employed for industrial use. Lastly, the NCYC495 strain (CBS1976; ATAA14754, NRLL-Y-1798) is commonly used in the laboratory and was isolated at Florida from concentrated orange juice (Wickerham, 1951). Phylogenetic analysis showed that *H. polymorpha* appears to be two different species: *Ogataea polymorpha and Ogataea parapolymorpha* (Kurtzman and Robnett, 2010; Suh and Zhou, 2010). The strain NCYC495 and CBS4732 are closely related and renamed as *O. polymorpha*, whereas DL-1 strain is phylogenetically distant and reclassified as *O. parapolymorpha*. To avoid misunderstanding, the nomenclature *H. polymorpha* will be used in this review once both species share all characteristics elucidated in Figure 1.

Various studies focused on genetically modifying *H. polymorpha* strains for the production of several recombinant proteins (Gellissen et al., 1992; Hollenberg and Gellissen, 1997; Stöckmann et al., 2009). Later on, the advances in genomic-editing tools, optimization of transformation and cultivation protocols have led to the industrial development of *H. polymorpha*-based processes for the production of various pharmaceuticals. Currently, three commercially available Hepatitis B vaccines are produced using antigens derived from fermentative processes with *H. polymorpha:* HepavaxGene^®^ (Johnson & Johnson), Gen Vax B^®^ (Serum Institute of India) and Biovac-B^®^ (Wockhardt) (http://www.dynavax.com/about-us/dynavax-gmbh/). Moreover, biopharmaceuticals successfully produced in this yeast and already available in the market included hirudin (Thrombexx®, Rhein Minapharm), insulin (Wosulin®, Wockardt) and IFNa-2a Reiferon® (Rhein Minapharm) (Gellissen et al., 2005).

It is noteworthy that the last published review of bioprocess development at *H. polymorpha* was nearly 10 years ago (Stöckmann et al., 2009). Thus, this review brings up to date strategies and examples of using this yeast as a host for recombinant protein production. The focus will be given on the studies developed in the last decade and are summarized in Table 1. The relevance of this yeast for the production of recombinant proteins, especially those for human welfare, justifies this literature update. Besides, newly genomic tools developed in the past years which have improved genetic manipulation of *H. polymorpha* are also discussed.

**Table 1:** Recombinant proteins produced in the last decade using *H. polymorpha* as host.

**Protein**	**Maximum production**	**Promoter**	**Utilized carbon source**	**References**
Human serum albumin (HSA)	5.8 g/L	*MOX*	Glycerol/Methanol	Youn et al., 2010
Heat shock protein gp96	≈150 mg/L	*FMD*	Methanol	Li et al., 2011
Ferritin (FTH1)	1.9 g/L	*FMD*	Glycerol/Methanol	Eilert et al., 2012
Bacteriocin enterocin A (EntA)	4.8 μg/mL	*TEF1* (*Arxula adeninivorans*)	Glucose	Borrero et al., 2012
Granulocyte colony stimulating factor (GCSF)	ND	*FMD*	Methanol	Talebkhan et al., 2016
Streptavidin (SAV)	≈751 mg/L	*FMD*	Methanol	Wetzel et al., 2016
Human parathyroid hormone (PTH) fragment 1–34	150 mg/L	*FMD*	Glycerol/Methanol	Mueller et al., 2013
Penicillin	1.1 μg/mL	*MOX*	Glucose/Methanol	Gidijala et al., 2009
Human papilomavirus 16 L1 Protein (HPV16L1)	78.6 mg/L	*MOX*	Methanol	Li et al., 2009
HPV type 16 L1-L2 chimeric protein (SAF)	132.10 mg/L	*MOX*	Methanol	Bredell et al., 2018
Rabies virus glycoprotein (RVG)	14.6 mg/L	*FMD*	Glycerol	Qian et al., 2013
Hepatitis B virus PreS2-S antigen	250 mg/L	*MOX*	Methanol	Xu et al., 2014
Human papillomavirus Type 52 L1 Protein (HPV 52 L1)	ND	*MOX*	Methanol	Liu et al., 2015
Rotavirus VP6 protein (RV VP6)	3350.71 mg/L	*MOX*	Methanol	Bredell et al., 2016
Hepatitis E virus-like particles (HEV VLPs)	1.0 g/L	*MOX*	Methanol	Su et al., 2017
Uricase from *Candida utilis*	52.3 U/mL	*MOX*	Methanol	Chen et al., 2008
Lipase from *Yarrowia lipolytica* (YlLip11)	1,144 U/L	*TEF1* (*Arxula adeninivorans*)	Glucose	Kumari et al., 2015
T4 lysozyme	0.49 g/L	*Not used*	Glycerol	Wang et al., 2011
Staphylokinase (SAK)	1,212 mg/L	*FMD*	Glycerol/Methanol	Moussa et al., 2012

*ND, Not Determined*.

## Why Use *H. polymorpha* as Host for Heterologous Expression?

The advantages of *H. polymorpha* for industrial processes comprise high-cell-density fermentation, capacity to utilize low-cost substrates, an established defined synthetic media, status GRAS (Generally Regarded As Safe) and consolidated strategies for cultivation in bioreactors (Jenzelewski, 2002). This yeast features genome-editing tools available for genetic manipulation (Figure 1A). An efficient protocol for transformation by electroporation has been described previously (Faber et al., 1994) as well as protocols for transforming protoplast ((Tikhomirova et al., 1988)). Among them, the electroporation method is more efficient than the protoplast, yielding 1.7 × 10^6^/μg plasmid DNA vs. 2 to 3 × l0^4^/μg DNA. The lithium acetate-dimethyl sulfoxide method has also been used tested (Sohn et al., 1999; Heo et al., 2003; Kim et al., 2015b). Furthermore, a method using nanoscale carriers for DNA delivery was employed for the transformation of *H. polymorpha* with twice efficiency of those obtained by electroporation and 15-fold for LiAc/DMSO method (Filyak et al., 2013). Moreover, three independent research groups have recently developed the CRISPR/Cas9 genome-editing tool for *H. polymorpha* (Numamoto et al., 2017; Juergens et al., 2018; Wang et al., 2018). Finally, the three strains of *H. polymorpha* had its genome sequenced, DL-1 (Ravin et al., 2013), NCYC495 (Riley et al., 2016), and CBS4732 (Ramezani-Rad et al., 2003).

Strategies for heterologous protein production in *H. polymorpha* take advantage of the yeast ability to grow in the presence of methanol. The methanol inducible promoters, formate dehydrogenase (*FMD*), and methanol oxidase (*MOX*) are the most utilized in genetic engineering strategies as it can be seen in Table 1. Shifting to methanol-feed led to upregulation of genes involved in its catabolism, for example the *FMD* gene was approximately 350-fold upregulated, while the *MOX* and *DHAS* genes were 17.3 and 19-fold upregulated when compared to growth on glucose (van Zutphen et al., 2010). Although an upregulation does not necessarily indicate a high promoter activity, other studies have shown that in the presence of methanol the *MOX* and *FMD* promoters present an enhanced activity (Amuel et al., 2000; Suppi et al., 2013).

The methanol-inducible promoters are not present only in *H. polymorpha* but in all methylotrophic yeasts. For instance, the well-known yeast *Pichia pastoris* (recently renamed as *Komagataella* sp.) is the yeast host more utilized for recombinant protein production. Although *P. pastoris* also has methanol-inducible promoters, the advantage of using *H. polymorpha* is that some of them are derepressed in the presence of glycerol which is less pronounced in *P. pastoris* (60–70% vs. 2–4% of induced levels) (Hartner and Glieder, 2006; Vogl and Glieder, 2013). In a comparative study, The Kunitz-type protease inhibitor (*KPI*) encoding gene was inserted in *H. polymorpha* and *P. pastoris* under the control of the alcohol oxidase, *AOX1* promoter (Raschke et al., 1996). For both yeasts, no mRNA encoding for KPI was detected when the cells were cultured in glucose as the carbon source but were abundant when induced by methanol. However, when cells grew in glycerol, it was possible to detect KPI only in *H. polymorpha*. Therefore, the derepression of methanol inducible promoters is a favorable feature of *H. polymorpha* over other methylotrophic yeasts.

Additionally, *H. polymorpha* is thermotolerant while *P. pastoris* is not (Figure 1B). The increase in temperature does not imply a higher yield of the recombinant protein but is relevant in industrial processes since it reduces microbial contamination and cooling costs (Abdel-Banat et al., 2010). Also, higher temperatures facilitate the implementation of Simultaneous Saccharification and Fermentation (SSF) since the thermal resistance allows the utilization of thermophilic hydrolases (Voronovsky et al., 2009). The *EGII* gene encoding endoglucanase II from *Trichoderma reesei* was produced in both methylotrophic yeasts, and the recombinant proteins were characterized. Although the secreted enzymes showed optimum activity at the same temperature (75°C), the one produced by *H. polymorpha* was shown to be more thermostable while remaining active after incubation at high temperatures (60–80°C) (Akbarzadeh et al., 2013).

If the recombinant protein is to be produced under the control of a methanol inducible promoter, the cultivation in the bioreactor is performed in a two-step. Initially, the growth phase is performed using glucose or glycerol as a carbon source in batch cultures. Then, the induction phase is accomplished by feeding the bioreactor with methanol or methanol/glycerol mixtures that can be added continuously or in pulses. For instance, the production of ferritin in *H. polymorpha* using a methanol and glycerol mixture (4:1) during the induction phase resulted in 1.9 g L of the recombinant protein (Table 1) (Eilert et al., 2012). In the case of the rabies virus glycoprotein production, only glycerol was employed during the induction phase (Qian et al., 2013). Despite the low levels achieved (14.6 mg/L), this is an example that glycerol can be used in both the growth and induction phases of this yeast. Although these two strategies are feasible to control recombinant protein production, the most common strategy employed is induction using only methanol 0.5–1% (Table 1).

A disadvantage of using such methanol inducible promoters is their repression caused by the presence of glucose in the media, although the derepression has already been reported (Mayer et al., 1999). In this point of view, *H. polymorpha* strains deficient in glucose repression represent alternative platforms for recombinant protein production (Krasovska et al., 2007). These mutants have a knock out at the *GCR1* gene that encodes a hexose transporter with altered activity leading to several alterations in the cell metabolism, including the derepression of methanol-related genes in the presence of glucose (Stasyk et al., 2004). Thus, the methanol-induced promoters controlling the recombinant protein production might be induced by either methanol or glucose. The *H. polymorpha gcr1* mutants are commonly utilized as the bio-elements for Yeast-based biosensing. Some practical examples of these biosensors utilization include the detection of L-lactate (Smutok et al., 2007), urate (Dmytruk et al., 2011), formaldehyde (Sigawi et al., 2014), and D-lactate (Smutok et al., 2018).

Some *H. polymorpha*-based platforms utilize the strong constitutive promoter *GAP* instead of methanol-inducible ones (Heo et al., 2003). The use of this promoter for the construction of recombinant strains, as well as *gcr1* mutants, enables the production of proteins without the addition of methanol. Since this molecule is flammable and toxic, avoiding its use can be advantageous. Indeed, several studies developing *H. polymorpha* strains for bioethanol production utilize genes under the control of the *GAP* promoter (Kurylenko et al., 2014, 2018). Examples of the utilization of the *H. polymorpha GAP* promoter included the development of thermotolerant strains, improvement of xylose utilization and those capable of Simultaneous Saccharification and Fermentation (SSF) as recently reviewed (Dmytruk et al., 2017).

Another advantage of using *H. polymorpha* as a host for recombinant protein production is its glycosylation pattern (Figure 1E). The yeasts frequently hyperglycosylate recombinant proteins. However, the intensity and type of sugar added dependent on both the organism and the sequence of the heterologous protein. The production of a recombinant glucose oxidase of *Aspergillus niger* was attempted using both yeasts *H. polymorpha* and *S. cerevisiae* (Kim et al., 2004). It has been shown that in *H. polymorpha* 27% less glycosylation was observed. Also, antibody anti-α1,3-mannose did not recognize the protein produced by *H. polymorpha* but was positive for that originated from *S. cerevisiae* which indicates that in *H. polymorpha* the recombinant protein was not immunogenic (Ballou, 1990). In the following years, efforts were made to develop engineered strains with human-pattern glycosylation (Kim et al., 2006; Oh et al., 2008; Cheon et al., 2012). These strains lack essential genes which encode enzymes for hypermannosylation pathways such as α-1,6-mannosyltransferase (Δ*hpoch1*) and dolichyl-phosphate-mannose dependent α-1,3-mannosyltransferase (Δ*hpalg3*) beside their has the human gene encoding β-1,2-N-acetylglucosaminyltransferase I (*GNTI*). The null mutants were able to produce human hybrid-type N-glycans (Cheon et al., 2012). Therefore, all the knowledge acquired about the physiology, metabolism, and genetics of this yeast enables its utilization as a host for heterologous protein production.

## The *H. polymorpha* Genetic Engineering Tools

The viability of tools for fast and precise genome edition is crucial for the development of the expression platforms. Some methods for the genetic manipulation of *H. polymorpha* have already been described, both for gene introduction and deletion (Figure 1A). It has been previously reported that episomal plasmids are mitotically unstable in *H. polymorpha* (Bogdanova et al., 1995, 2000) and consequently are not suitable for the development of industrial strains. Episomal plasmids contain the *H. polymorpha* autonomous replicating sequences *(HARS*) derived from subtelomeric regions (Sohn et al., 1996). Nevertheless, the *HARS* sequences do not guarantee stability for circular plasmids. Thus, the prolonged incubation in selective medium forces the plasmid integration usually in the respective subtelomeric locus (Kim et al., 2003). Hence, the integrative plasmids are most suitable for genetic manipulation of *H. polymorpha*. Depending on the locus of integration it is possible to reach between 1 up to 100 copies into the genome (Agaphonov et al., 1999).

The *H. polymorpha* integration plasmids (pHIP) series have several promoters and selective markers (For detailed information see (Saraya et al., 2012). They show an easy terminology in which the letter indicates the selective marker and the number indicates the promoter. For example, the pHIPH15, the “HIP” means “*Hansenula* Integration plasmid” while the letter stands for the selective marker, in this example hygromycin. The number indicates the promoter utilized in the plasmid and “15” is for *DHAS*. There are fifteen *H. polymorpha* promoters available in the pHIP series (https://www.rug.nl/research/molecular-cell-biology/research/the-hansenula-polymorpha-expression-system), and the site-specific integration is driven by linearization in the promoter region of the plasmid. For selection, auxotrophic and dominant markers can be used. They include *Sc LEU2, Hp URA3, Hp ADE11, Hp MET6, Sh-ble* (Zeocine), *Sn-nat1* (Nourseothricin), *Kp-hph* (Hygromycin B), and *Tn-KanMX* (G418/Geneticin) (Saraya et al., 2012). Another possibility for the introduction of an exogenous gene in *H. polymorpha* is the wide-range vector CoMed™ system (Böer et al., 2007). This vector was designed to fit in many species of yeast, enabling to save time and effort during the cloning procedure. The vector contains *ARS* and rDNA sequences that drive the integration of the plasmid into *H. polymorpha* genome. Besides, it was constructed in modules flanked by recognizing sites of restriction enzymes that allows the exchange of expression cassettes and selective markers.

Gene deletion in *H. polymorpha* is reached through the construction of cassettes containing a homologous region of the gene to be deleted flanking 5′ and 3′ of the target locus. Usually, the cassette has an antibiotic as a selection marker such as zeocin or hygromycin. Although there are different approaches for gene deletion in *H. polymorpha*, the disruption frequencies are of approximately 35% with homology arms ranging from 500 to 1,000 bp (Table 2). The deletion cassettes can be constructed by single-step PCR as an adaptation of the protocol previously utilized in *S. cerevisiae* (Manivasakam et al., 1995). A one-step mediated-PCR method for gene disruption was also previously reported (Gonzalez, 1999). As a proof concept, the gene *YNR1* of *H. polymorpha* strain NCYC495 (*ura3*) was disrupted utilizing a construct bearing the *URA3* auxotrophic marker flanked by homologous regions to the target gene. The homologous arms 5‘ and 3‘ tested varied between 25 and 1,000 bp in size with the best results obtained with the larger homology (35%). Similar results were observed in the disruption of *MOX* gene (36%) with 1,000/1,000 homologous arm size (Gonzalez, 1999) (Table 2).

**Table 2:** Most common techniques used for gene deletion in *H. polymorpha*.

**Target locus**	**Strain**	**Frequency of Deletion %**	**Method**	**Homologous arm size (5^′^/3^′^) bp**	**References**
*YNR1*	NCYC495	35	Deletion cassette	1,000/1,000	Gonzalez, 1999
*MOX*	NCYC495	36	Deletion cassette	1,000/1,000	
*MOX*	NCYC495 Δ*yku80*	88	Deletion cassette	245/247	Saraya et al., 2012
*MOX*	NCYC495	31	Deletion cassette	245/247	
*ALG3*	NCYC495	35	Deletion cassette	491/520	Qian et al., 2009
*ALG3*	NCYC495	76	Co-transformation with single-stranded DNA	491/520	
*ALG3*	NCYC495	19	Deletion cassette	~250/250	
*ALG3*	NCYC495	33	Co-transformation with single-stranded DNA	~250/250	

In another study, the deletion cassettes were designed containing flanking regions with different lengths for targeting *MOX* locus. In size, the homologous arms were tested between 30 and 250 bp. The deletion frequency for fragments of up to 50 bp was only ±12%. The larger homologous arm tested showed an incidence of 31% (Table 2). Another strategy adopted was the deletion of *ku80* aiming at increasing the deletion frequency (Saraya et al., 2012). This gene deletion increases the repair by homologous recombination (HR) instead of non-homologous end joining (NHEJ), ensuring site-specific integration. The Δ*yku80* strain was generated by replacing this gene with the *URA3* gene. For cassettes designed to target in *MOX* gene with Hygromycin B as selection marker and flanking regions approximately 250 bp, the deletion efficiency was 88% in Δ*yku80* strain vs. 31% in the wild-type (Table 2).

The limited number of available markers can impair multiple gene insertion or deletion. Therefore, recycling the selective markers using the Cre–*loxP* recombination technique has been applied in *H. polymorpha* (Qian et al., 2009; Agaphonov and Alexandrov, 2014). The gene *PMC1* encoding for the protein Calcium-transporting ATPase 2 was disrupted in the strain DL1-L (*leu2*) using auxotrophic marker *LEU2*. The *MOX* promoter drives the *CRE* gene expression, so when methanol was added into the cultivation medium, the cassette contained the marker *LEU2* was excised from the genome. Furthermore, these clones were unable to grow in the presence of CaCl_2_ corroborating the *PMC1* deletion. The combination of different approaches was utilized to increase the efficiency for gene deletion in *H. polymorpha* NCYC495 (Qian et al., 2009). The knock-out system uses a sticky-end polymerase chain reaction method for the construction of deletion cassettes, LiAc/single-stranded (SS)-DNA/PEG for cell transformation and l*oxP*-flanked selective markers for multiple deletions. The main advantage of this approach is the use of single-stranded DNA for co-transformation with deletion cassettes. Two genes were targeted, *URA5* and *ALG3*, encoding the orotate-phosphoribosyl transferase (OPRTase) and alpha-1,3-mannosyltransferase, respectively. The cassette bearing *loxP*-*kanMX*-*loxP* with ~500 bp or ~250 bp homologous arms were co-transformed with and without single-stranded DNAs. For gene *ALG3*, the frequency of homologous recombination increased from 19 to 33% with ~250 bp homologous arms in the presence of single-stranded DNA (Table 2). When 500 bp homology regions were utilized, the frequency raised from 35 to 76% for co-transformation with single-stranded DNA (Table 2). The same pattern was observed for the *URA5* locus, for 250 bp 17% and 500 bp 31% without co-transformation while in the presence of single-stranded DNA were 32 and 73%, respectively (Qian et al., 2009).

At present, the CRISPR/Cas9 technologies have been applied in various organisms aiming at genome edition by the promise of being more rapid and low cost than previously available technologies [see details in Donohoue et al. (2018)]. Three studies already reported the use of CRISPR/Cas9 in *H. polymorpha* (Table 3). In the first one, the locus *ADE2* in the NCYC495 was targeted due to the red-phenotype that can be easily visualized upon successful deletion (Numamoto et al., 2017). For that, an episomal plasmid expressing both Cas9 and gRNA guided by the promoters *ScTEF1* and *OpSNR6*, respectively was introduced in *H. polymorpha*. In the absence of the *ADE2* DNA donor, the deletion frequency was around 10^−3^. When a DNA donor with 60/60 bp of homologous arms for *ADE2* locus was co-transformed with Cas9 and gRNA_*ADE*2_, it increased the efficiency by up to 47%. To evaluate the system efficiency in another locus, the loci *ADE8* and *PHO8* were disrupted resulting in 0.36 and 0.08% efficiencies, respectively. Aiming at improving the CRISPR/Cas9 system in this yeast, the promoters guiding Cas9 and gRNA were substituted for *OpTDH3* and *tRNA* promoters, respectively. The cells were then transformed with these plasmids into two combinations: *ScTEF1* (Cas9) and *tRNA* (gRNA_*ADE*2_) promoters and *OpTDH3* (Cas9) and *tRNA* (gRNA_*ADE*2_) promoters. Without the DNA donor, the first combination reached a frequency of 38% whiles the second one 45% for the locus *ADE2*. Finally, the improved system in that *OpTHD3* promoter guide the Cas9 expression and the *tRNA* promoter the gRNA expression was used for deletion of three genes from phosphate signal transduction (PHO) pathway, *PHO1, PHO11*, and *PHO84*. In this case, more than one gRNA was tested for each locus, and no DNA donor was utilized. Four gRNAs were designed for the *PHO1* gene, however, in 2 of them, the disruption efficiency was 0% while for another two 50, and 71% were observed. For the locus *PHO11*, one of three showed 0% of disruption and 17 and 30% for the other gRNAs. The last locus targeted *PHO84* obtained the same frequency of approximately 67% for both two gRNAs utilized (Numamoto et al., 2017).

**Table 3:** CRISPR/Cas9 systems available for genomic editing in *H. polymoprha*.

**Strain**	**Cas9 Promoter**	**Cas9 origin**	**sgRNA Promoter**	**gRNA tested per target**	**Cas9 and gRNA in the same plasmid?**	**Plasmid type**	**target locus**	**% deletion efficiency**	**DNA donor (5^′^/3^′^)bp**	**Reference**
NCYC495	*TEF1* (*S. cerevisiae*)	*Homo sapiens*	*SNR6* (*H. polymorpha*)	1	Yes	Episomal	*ADE2*	10^−3^	No	Numamoto et al., 2017
	*TEF1* (*S. cerevisiae*)	*Homo sapiens*	*SNR6* (*H. polymorpha*)	1	Yes	Episomal	*ADE2*	47	Yes (60/60)bp	
	*TEF1* (*S. cerevisiae*)	*Homo sapiens*	*SNR6* (*H. polymorpha*)	1	Yes	Episomal	*ADE8*	0.36	No	
	*TEF1* (*S. cerevisiae*)	*Homo sapiens*	*SNR6* (*H. polymorpha*)	1	Yes	Episomal	*PHO8*	0.08	No	
	*TEF1* (*S. cerevisiae*)	*Homo sapiens*	*tRNA^*CUG*^*(*H. polymorpha)*	1	Yes	Episomal	*ADE2*	38	No	
	*TDH3* (*H. polymorpha*)	*Homo sapiens*	*tRNA^*CUG*^*(*H. polymorpha)*	1	Yes	Episomal	*ADE2*	45	No	
	*TDH3* (*H. polymorpha*)	*Homo sapiens*	*tRNA^*CUG*^*(*H. polymorpha)*	4	Yes	Episomal	*PHO1*	71	No	
	*TDH3* (*H. polymorpha*)	*Homo sapiens*	*tRNA^*CUG*^*(*H. polymorpha)*	3	Yes	Episomal	*PHO11*	30	No	
	*TDH3* (*H. polymorpha*)	*Homo sapiens*	*tRNA^*CUG*^*(*H. polymorpha)*	2	Yes	Episomal	*PHO84*	67	No	
DL-1	*TEF1* (*Arxula adeninivorans*)	iCas9	*TDH3* (*S. cerevisiae*)	1	Yes	Episomal	*ADE2*	63 ± 2	ND	Juergens et al., 2018
	*TEF1* (*Arxula adeninivorans*)	iCas9	*TDH3* (*S. cerevisiae*)	1	Yes	Episomal	*ADE2* and *YNR1*	2-5	ND	
CBS4732	*TEF1* (*Arxula adeninivorans*)	iCas9	*TDH3* (*S. cerevisiae*)	1	Yes	Episomal	*ADE2*	9 ± 1	ND	
Laboratory strain CGMCC7.89	*TEF1* (*S. cerevisiae*)	iCas9	*SNR52* (*S. cerevisiae*)	1	No	Integrative	*LEU2*	58.33 ± 7.22	Yes (1,000/1,000)bp	Wang et al., 2018
	*TEF1* (*S. cerevisiae*)	iCas9	*SNR52* (*S. cerevisiae*)	1	No	Integrative	*URA3*	65.28 ± 2.41	Yes (1,000/1,000)bp	
	*TEF1* (*S. cerevisiae*)	iCas9	*SNR52* (*S. cerevisiae*)	1	No	Integrative	*ADE2*	62.18	Yes (1,000/1,000)bp	
	*TEF1* (*S. cerevisiae*)	iCas9	*SNR52* (*S. cerevisiae*)	1	No	Integrative	*ADE2*	37.18	Yes (500/500)bp	
	*TEF1* (*S. cerevisiae*)	iCas9	*SNR52* (*S. cerevisiae*)	1	No	Integrative	*URA3, LEU2* and *HIS3*	23.61	Yes (1,000/1,000)bp	
	*MOX* (*H. polymorpha*)	iCas9	*SNR52* (*S. cerevisiae*)	1	No	Integrative	*rDNA*	75	Yes (1,000/1,000)bp	

ND* No difference was observed in deletion efficiency with or without HR donor.

In another study, the CRISPR/Cas9 was used for genome edition in both *Kluyveromyces lactis* and *Ogataea* sp. In the last one, the utilized strains were CBS4732 and DL-1 (Juergens et al., 2018). The *AaTEF1* promoter was used to guide a Cas9 variant with higher activity, known as improved Cas9 (iCas9) (Bao et al., 2015). The promoter *ScTDH3* was used to control the gRNA expression. The *ADE2* locus also was disrupted with the same gRNA for both strains *O. polymorpha* (CBS 4732) and *O. parapolymorpha* (DL-1). The disruption frequencies varied between the two species which may indicate that even in phylogenetically close species the efficiency of the CRISPR/Cas9 system may require adjustments. The red phenotype, expected for colonies whose *ADE2* was deleted, just appeared after prolonged incubation time with disruption rates of 9% for *O. polymorpha* while *O. parapolymorpha* showed ~61 and 63% after 96 and 192 h of incubation, respectively. Surprisingly, the utilization of marker-free DNA donor did not affect the deletion efficiency in this case. Also, a multiple gene editing for multi-locus targeting was employed in this study. A plasmid carrying gRNAs targeting the loci *ADE2* and *YNR1* was utilized simultaneously for gene disruption. The gRNAs were placed in a tandem array spaced by a 20-bp linker. Although the deletion frequencies reached values between only 2 and 5%, this was the first report of multiple genes editing in *H. polymorpha* through CRISPR/Cas9 (Juergens et al., 2018).

Recently, an efficiently multiplex system was employed using a laboratory strain CGMCC7.89 (from the China General Microbiological Culture Collection Center) as host (Table 3). The plasmids carrying the Cas9 and gRNA were inserted into the genome to ensure their stability through up- and downstream homologous arms (~1.5 kb). The Cas9 was integrated into the *MET2* locus while and the gRNA into the *ADE2* locus. The positive clones were selected by the respective auxotrophies. All transformations were performed with the presence of donor DNA consisting of a fragment containing the up- and downstream region of the target gene amplified from the *H. polymorpha* genome. Initially, the *LEU2* and *URA3* were targeted to test the CRISPR/Cas9 system. Successful deletion occurred with 58.33 ± 7.22% for *LEU2* and 65.28 ± 2.41% for *URA3*. The effect of the size of the homology arms was tested using the *ADE2* locus flanking regions from 50 to 1,000 bp. When 50 bp was utilized, the efficiency was 0%, for 500 and 1,000 bp the values obtained were 37.18 and 62.18%, respectively. Therefore, all editing templates used for subsequent experiments had 1,000/1,000 bp of homology. These results corroborate the hypothesis that for *H. polymorpha* at least 500 bp are required to ensure deletion efficiency. The competence for multiple simultaneous deletions was verified with an efficiency of 23.61% for three loci *URA3, LEU2*, and *HIS3*. Next, a template containing a *gfpmut3a* expression cassette flanked by up- and downstream homologous arms of the three loci, *URA3, LEU2*, and *HIS3*, were used separately as DNA donor to prove that gene insertion was feasible using CRISPR/Cas9. The efficiency of integration observed reached 66.7%, 66.7% and 62.50% for *HIS3, URA3*, and *LEU2*, respectively. After that, the *gfpmut3a* was replaced by genes encoding the proteins within the resveratrol metabolic pathway that resulted in maximum production of 4.7 mg/L. The effect of gene copy number required for resveratrol synthesis was tested by multiple integrations into rDNA regions using CRISPR/Cas9. In this strategy, the *TEF1* promoter that was driving the expression of the CAS9 encoding gene was replaced by the methanol inducible *MOX*. In the presence of the *gfpmut3a* cassette with homologous arms, its insertion in the rDNA region occurred with a frequency of 75% and 11 copies of *gfpmut3a* were quantified in one of the studied clones. Finally, this approach was used to introduce the three genes that enable resveratrol synthesis in this yeast. The maximum resveratrol production was 97.23 ± 4.84 mg/L in a strain containing nine copies of each gene (Wang et al., 2018).

## Examples of Recombinant Proteins in *H. polymorpha*

The latest recombinant proteins that utilize *H. polymorpha* as host are summarized in Table 1 although, up to this moment, none of the listed examples were performed at industrial scale. The first heterologous protein produced in *H. polymorpha* was the surface antigens from hepatitis B virus (Janowicz et al., 1991). Furthermore, *H. polymorpha*, has been extensively used for the production of Virus-like particle (VLP), which are viral proteins that can be used for the development of vaccines (Kumar and Kumar, 2019). Only in the last decade, *H. polymorpha* was used to produce VLPs for the hepatitis B virus (Li et al., 2011; Xu et al., 2014), hepatitis C virus (He et al., 2008), hepatitis E virus (Su et al., 2017), Human papillomavirus (Li et al., 2009; Liu et al., 2015; Bredell et al., 2018), rabies virus (Qian et al., 2013), and rotavirus (Bredell et al., 2016). Recently, a chimeric VLP was development for *H. polymorpha* (Wetzel et al., 2018). In this type of platform, viral proteins are produced heterologously as chimeric proteins containing antigens from different viruses, allowing the simultaneous production of different VLPs. For *H. polymorpha*, the developed chimeric VLPs contained viral proteins from bovine viral diarrhea virus, the classical swine fever virus, the feline leukemia virus, and the west Nile virus, using as scaffold the small surface protein of the duck hepatitis B virus (Wetzel et al., 2018).

Further examples of recombinant proteins using *H. polymorpha* as host include the human Parathyroid Hormone (PTH) and Staphylokinase (SAK). Here it is summarized how the recombinant strain was constructed, the process was optimized and scaled-up to 80 liters (Moussa et al., 2012; Mueller et al., 2013). The PTH is a hormone secreted by the parathyroid gland responsible for calcium homeostasis in the blood and osteogenesis. It is a glycoprotein, but it has already been shown that non-glycosylation does not affect its biological activity (Bisello et al., 1996). The N-terminal 1–34 fragment of this protein has an essential function as a receptor binding region and is employed for the treatment of osteoporosis (Cho et al., 2018). The integrative plasmid pFPMT-MFα containing the coding region for the 1–34 fragment of PTH fused with MFα signal was inserted into Δ*yps7* strain (deficient protease YPS7). The *FMD* promoter controlled the production of PTH, and the plasmid carried a *HARS* sequence for genomic integration (Kim et al., 2003). Positive clones for PTH production were selected for medium optimization on a micro-plate scale.

Using micro-scale assays, it has been possible to evaluate the influence of culture media on the PTH titers. At this stage, all standard complex media (YPD, YPG, YNB) in addition to two synthetic media Syn 6 and Syn 6-cp (supplemented with citrate and peptone) were tested. The best PTH concentration (~40 mg/L) was achieved with the Syn6-cp medium. After that, the influence of three peptones (soy, wheat, and potato) in a 100 mL shake scale was investigated with the highest production of the recombinant protein (9.6 mg/L) was achieved using wheat peptone. Next step was performed in 250 mL baffled flasks with 100 mL of working volume in fed-batch mode. Manual applications after 20, 24, 42, 46, and 50 h of inducing-mixture containing a methanol/glycerol (1:1) and wheat peptone resulted in 25.4 mg/L of PTH. In the following steps, 300-mL bioreactors were utilized to test these parameters under controlled fermentation conditions. Cell cultures were pre-grown in glycerol 3% for 24 h. After that, the induction phase was performed with methanol pulsed every 6 h during 24 h leading to the production of 68.3 mg/L of PTH. A similar result was obtained in 2 L reactors (67 mg/L) using the same strategy. Finally, 8 L and 80 L fermentation were performed using the optimum conditions. For that, the partial oxygen pressure (pO2) was set to 30%. The feeding strategy was changed from pulse-wise to feeding scheduled realized at a constant rate ranging from 20 to 60 g/L/h for 52 h. Using this fermentation strategy was obtained 120 mg/L and 150 mg/L PTH in 8 and 80 L, respectively (Mueller et al., 2013).

A similar approach has been used for the heterologous production of Staphylokinase (SAK), a biopharmaceutical with pro-fibrinolytic activity (Moussa et al., 2012). Recombinant strains producing an SAK THR164 variant (ThromboGenics NV) were developed and the process was scaled-up from micro-titer plates to 80 L. Two isoforms encoding the SAK protein, rSAK-1 and rSAK-2, were placed under control of the *FMD* promoter, in addition to containing the MFα1 sequence for secretion. The rSAK-2 had a substitution of the Thr-30 amino acid residue for an alanine. This is a recognition site for N-glycosylation and its mutation showed to affect the glycosylation pattern of the produced protein. The final plasmids bearing *URA3* auxotrophic marker and containing the genes encoding rSAK-1 and rSAK-2 were transformed into the strain RB11, a uracil-auxotrophic variant of CBS4732. After the selection, the positives clones were tested for rSAK-1 and rSAK-2 production in shake flasks using YPG (glycerol). The Western blot assay demonstrated that r-SAK1 and not r-SAK-2 was glycosylated. Taking into account that glycosylated SAK shows reduction in enzymatic activity, only strains producing rSAK-2 were further utilized for process optimization.

The micro-plate assays were employed to evaluate the pH, medium and feeding strategy effects on rSAK-2 production using SYN6 medium as basis. The screening of pHs ranging 4–8, twenty different peptones and two feeding strategies indicated that the highest production of rSAK-2 (90 mg/L) occurred in pH 6.5 with SYN6 supplemented with wheat peptone and induced by methanol. Further medium optimization was realized in 500 mL shake flasks with a working volume of 100 mL. At this stage, variations of synthetic medium SYN6 were tested. The removal of trace elements and vitamins of SYN6 yielded 200 mg/L of rSAK-2 after 48 h of fermentation, the best production achieved in shake flasks. This medium was designed as SYN6.46d. After that, the process was transferred to 300 mL bioreactors where two feeding solutions were evaluated. The first one, named as FEED_1, contained 20% yeast extract (w/v), 10% peptone (w/v), 5% glycerol (w/v), and 10% methanol (w/v). While FEED_2 in turn was composed by 20% peptone (w/v), 10% glycerol (w/v), 20% yeast extract (w/v) and 10% methanol (w/v). In constant feeding mode (4 mL of feeding solution per hour) using FEED_2, the yield of rSAK-2 was 423 mg/L at 48 h of fermentation, the highest amount reached so far. Finally two parameters were adjusted before scaling-up the process from 300 mL bioreactors. The air flow was setting to 2 L/min instead 0.5 L/min and the stirring speed was changed from 500 rpm to 800 rpm. Lastly, fermentations using the medium SYN6.46d in a constant fed-batch mode with the feeding solution FEED_2 were performed at bioreactors of 2, 8, and 80 L that yielded rSAK-2 amounts of 1,212, 1,081, and 1,109 mg/L, respectively (Moussa et al., 2012).

Although *H. polymorpha* is more frequently used as host for recombinant protein, the production of chemicals and fatty acids has also been described. The most extensive example discussed in the literature is the utilization of *H. polymorpha* to produce ethanol using xylose as carbon source. Up to now the best reported strain achieved 12.5 g/L using a strain where *CAT8* gene was disrupted. This gene encoding a transcriptional activator involved in the regulation of xylose metabolism in *H. polymorpha* (Ruchala et al., 2017). For an extensive review on this particular bioprocess the reader is directed to a recently published review (Dmytruk et al., 2017). Production of γ-linolenic acid was achieved in *H. polymorpha* by the introduction of *Mucor rouxii* Δ^6^-desaturase gene under control of the *MOX* promoter (Khongto et al., 2010). Despite the utilization of a methanol-inducible promoter, the maximum γ-linolenic acid titers, 697 mg/L, was reached when glycerol was used as the carbon source. For the production of 1,3-propandiol, all six genes necessary for its biosynthesis were transferred from *Klebsiella pneumoniae* (Hong et al., 2011). All inserted genes were present in one single plasmid under the control of the *GAP* promoter. The resulting strains produced 2.4 g/L and 0.8 g/L of 1,3-Propandiol using glucose and glycerol as substrate, respectively (Hong et al., 2011). Similarly, the insertion of four genes were introduced in *H. polymorpha* enabling the synthesis of 5-hydroxyectoine (Eilert et al., 2013). Nevertheless, in this study all genes were cloned in different plasmids, where each plasmid had upstream- and downstream regions amplified from the yeast genome to direct the integration and a unique selection marker. The resulting strain was able to achieve 2.8 g/L of 5-hydroxyectoine using methanol as the carbon source. Recently, CRISPR/Cas9 was utilized to introduce three genes (*TAL, 4CL*, and *STS*) required for the resveratrol synthesis in *H. polymorpha* (Wang et al., 2018). All genes were cloned into one expression cassette and integrated into the *rDNA* locus aiming at multiple copy integrations. The best producing strain contained 9 copies of each gene and was able to produce approximately 98 mg/L (Wang et al., 2018).

## Conclusions

The development of new cell factories for the production of heterologous proteins that are scarce is the primary challenge of the 21st century. Advances in molecular genetics and cultivation techniques drive the growing number of new expression platforms. Among these, the yeast *H. polymorpha* stand out as host due to the presence of strongly methanol-inducible such as *MOX* and *FMD* promoters, the glycosylation pattern compatible with human glycoproteins, the thermotolerance capacity and it is able to use different carbon sources (Figures 1A–E). Furthermore, various genetic engineering tools and transformation protocols are established in this yeast. Nevertheless, the low frequency of homologous recombination in *H. polymorpha* delays the strain construction step. Many efforts were made to bypass this problem: utilization of *ku80* strain, methods for construction of deletion cassettes and implementation of CRISPR/Cas9 technology.

Genome editing via CRISPR/Cas9 represents a powerful tool for genetic manipulation. It is possible to perform not only the deletion of endogenous genes from the organism but also the insertion of exogenous sequences into its genome. The three systems implemented in *H. polymorpha* allowed disruption or the introduction of exogenous genes. Furthermore, the utilization of episomal plasmids for CRISPR/Cas9 implementation in *H. polymorpha* required modifications into the initial strategy to enhance the deletion frequencies (Table 3). In both cases, adjustments to the developed approach increased the efficiency of the system: substitution of promoters (Numamoto et al., 2017) or prolonged incubation times to guarantee the activity of Cas9 (Juergens et al., 2018). Implementation of CRISPR/Cas9 through integrative plasmids guaranteed gene deletion rates >50% for different loci (Wang et al., 2018). Also, co-transformation with a DNA template to induce HR repair after slicing of Cas9 was more efficient than using only Cas9 and gRNA (Table 3). The CRISPR/Cas9 system was also efficient for the introduction of exogenous genes into *H. polymorpha* (Wang et al., 2018). It was possible to introduce a complete pathway for the synthesis of resveratrol in *H. polymorpha* in a single transformation event, representing a revolution in the genetic manipulation of this yeast. Thus, it is evident how the utilization of genome editing tools will reduce the time and cost of strain construction allowing a rapid introduction into the commercial scale.

Finally, the examples given for recombinant protein production can be extrapolated to the production of other molecules. Micro-scale cultivation is often used to optimize culture conditions by testing different mediums composition, pH and other factors that may affect the productivity of the desired protein. The best results are scaled up adding the feeding and oxygenation strategies. Altogether these features elucidated how *H. polymorpha* is a promising host for the establishment of various bioprocesses. This is reflected already by the number of products available in the market and by the pipeline of those that are in the optimization phase.

## Author Contributions

JM-N wrote the manuscript. AG prepared the figure. NP assisted with writing, editing, and finalizing the manuscript. All authors approved its publication.

### Conflict of Interest Statement

The authors declare that the research was conducted in the absence of any commercial or financial relationships that could be construed as a potential conflict of interest.
